# Supplementary material for: Mere presence of co-eater automatically shifts foraging tactics toward ‘Fast and Easy' food in humans
Source: R Soc Open Sci. 2020 Apr 1;7(4):200044. doi: 10.1098/rsos.200044 (PMC7211848; doi:10.1098/rsos.200044)
Supplement: Supplementary materials and methods [file rsos200044supp1.docx]

**Supplementary materials and methods for “Mere presence of co-eater automatically shifts foraging tactics toward “fast and easy” food in humans”**

Yukiko Ogura, Taku Masamoto and Tatsuya Kameda

## *Subjects*

The study was approved by, and carried out in accordance with the guidelines and regulations of, the ethical committee of the Department of Social Psychology of the University of Tokyo (IRB_SP2017_001). All subjects responded to an advertisement for a “potato chip tasting experiment”, and provided written informed consent, approved by the ethical committee, prior to the experiment.

Total of 64 students (34 males and 30 females) at the University of Tokyo were called to the tasting experiment, with either one or two subjects in each experimental slot. We randomly assigned the subjects to one of three conditions: *Solo*, *Invisible Pair* or *Visible Pair*. In the *Pair* conditions, two subjects who met for the first time at the experiment were sex-matched. Of those, one pair of males in the *Visible Pair* condition was excluded because they happened to know each other prior to the experiment. Data from one pair of males in the *Visible Pair* condition and one male subject in the *Solo* condition were also discarded due to technical problems. To screen for eating disorders, we asked each subject to (1) provide their height and weight so that we could calculate their body mass index (BMI) and (2) answer a Japanese version of the 26-item Eating Attitudes Test (EAT-26) [1,2] in the post-session questionnaire. According to predetermined exclusion criteria (BMI less than 17 and EAT-26 score greater than 15), no subject was excluded from the analysis. As a result, we had 59 subjects for analysis (5 male pairs and 5 female pairs each in the *Visible* and *Invisible* *Pair* conditions, and 9 males and 10 females in the *Solo* condition; Males: 21.79 ± 1.21 yrs, Females: 22.07 ± 3.47 yrs). In the post-session questionnaire, we also checked subjects’ handedness with a Japanese version of the FLANDERS ﻿handedness questionnaire [3,4], which showed that 54 subjects were right-handed, 2 subjects were mixed-handed, and 3 subjects were left-handed. We found no remarkable relationship between handedness and any of the behavioral responses.

## *Experimental procedures*

Each subject completed a written consent form and was provided with a portion of potato chips placed on a square white paper plate (20 × 20 cm). Each portion contained 30 grams of potato chips (Calbee Potato Chips Lightly Salted variety; Calbee Inc., Tokyo, Japan). Before the start of each session (3-minute tasting phase + rating phase), subjects were instructed that during the tasting phase they could eat any amount of the potato chips at their own pace. To measure the weight of potato chips per reach and reach frequencies of each subject, an electronic balance (EK-3000i, A&D Company, Limited, Tokyo, Japan) was pre-set under the food plate. The minimum weight measurement interval of the balance was 0.1 gram, and the temporal resolution for data acquisition was 0.1 second (i.e. 10 data points were acquired per second). A representative weight trajectory is shown in figure 1B. To prevent subjects from being aware of the weight measurement, the balance was covered by an opaque cardboard cover and a square paper food plate. Subjects were also videotaped during the session (with their consent). The experimenters left the room during the session, and an electronic chime cued the beginning and the end of the tasting phase.

After tasting, subjects were asked to evaluate the flavor of the potato chips on nine Likert-type continuous scales (0–100): (1) general deliciousness, (2) sweetness, (3) saltiness, (4) sourness, (5) bitterness, (6) umami, (7) strength, (8) texture, and (9) fillingness. We observed no significant difference in ratings across the three conditions (figure S1).

After the three tasting and rating sessions, subjects answered a post-session questionnaire that asked: (1) how much they cared about the presence of the other subject during tasting (in the *Pair* conditions only), (2) how hungry they were during tasting, and (3) the degree to which they were concerned about their body shape and diet-related behaviors (frequency of exercise, restrictions on diet quantity and quality). Subjects answered these items on Likert-type scales ranging from 0 to 100. As mentioned above, subjects also answered EAT-26 and FLANDERS handedness questionnaire. No questionnaire item predicted any of the behavioral responses examined in the study (table S5). All ratings in the questionnaire were conducted using an ﻿online form (Qualtrics; Qualtrics LLC, Utah, USA).

## *Procedures for assessing distribution of potato-chip weights*

We counted and weighed all 8,075 pieces of potato chips contained in a total of 100 bags of 60g each. The bags were purchased at three different stores to avoid all bags being from the same lot. Two of the authors (YO and TM) weighed 2,915 and 2,838 pieces, respectively. We divided the remaining 2,322 pieces of potato chips into 30 portions of 30 grams each and placed them on the same type of paper plate used in the tasting experiment. We recruited two naïve female subjects and asked them to weigh each piece from the plate (without eating any) and record the weight sequentially on a spreadsheet (figure 3A left). Each subject conducted the weighing experiment in a separate booth. When each subject finished measuring all of the pieces in a portion, we provided another 30g portion of potato chips. Each subject measured 15 portions (1,153 and 1,169 pieces). Their reach behaviors for weighing were measured in the same manner as in the tasting experiment.

## *Statistical analysis using Bayesian multilevel models for reach frequency and food intake amount*

To analyze the data, we used Bayesian generalized linear mixed modeling, with parameters estimated by the Bayesian MCMC (Markov chain Monte Carlo) method, rather than the maximum likelihood method.

We assumed a gamma distribution for the error structure of the data of reach frequency and food intake amount, considering that they took only non-negative values. *Λ*(*X*) (>0) was thus approximated by a gamma function (log link function) as

$\begin{aligned} \Lambda\left( X \right)=\exp\left( X \right)\#\left( SEQ Equation \backslash* ARABIC 1 \right) \end{aligned}$The full model is as follows:

$$\begin{aligned} X \sim Gamma\left( \begin{aligned} \beta_{0}+\beta_{1}\times sex+\beta_{2}\times condition+\beta_{3}\times session \\ + \beta_{4}\times sex:condition+\beta_{5}\times sex:session+\beta_{6}\times condition:session \\ +\beta_{7}\times sex:condition:session \\ +\frac{r_{pair}}{r_{individual}} \end{aligned} \right)\#\left( 2 \right) \end{aligned}$$

﻿﻿*Sex* is a categorical variable with male assigned to the baseline (0), and coefficient $\beta_{1}$ indicates the difference in females compared to males; a negative estimated value for $\beta_{1}$ indicates that the response variable *X* was smaller in females compared to males. *Condition* is a categorical variable with the *Solo* condition assigned to the baseline, and Coefficient $\beta_{2}$ indicates the difference in the *Visible Pair* or *Invisible Pair* condition compared to the *Solo* condition; a positive estimated value for $\beta_{2}$ indicates that the response variable *X* was larger in the condition compared to *Solo* condition. *Session* is a categorical variable with the first session assigned to the baseline, and coefficient $\beta_{3}$ indicates the difference in the remaining sessions compared to the first session; a positive estimated value for $\beta_{3}$ indicates that the response variable *X* was larger in the session compared to the first session. To examine the possibility that condition or saturation has an effect only on one sex, the interaction term was added. Coefficients $\beta_{4}{, \beta}_{5}, \beta_{6},\beta_{7}$ indicate the weights for the interactions *sex*×*condition*, *sex*×*session*, *condition*×*session*, and *sex*×*condition*×*session*, respectively. We included only interactions for which all components of the interaction were included in the model as main effects, because models that included interactions without main effects did not converge. The random intercepts for each pair and individual were denoted by $r_{pair}$ and $r_{individual}$, which represent variation between pairs and between individuals, respectively. For the error structure of the random intercept, we assumed a normal distribution with mean = 0.

The designed models were compared according to WAIC (Widely Applicable Information Criteria; [6]). Because WAIC varies depending on the random number seed of the MCMC, we ran 100 sampling runs per model, shifting the random number seed from 1 to 100. In each sampling run, we generated four chains of 5,000 samples. For each chain, the first half (2,500 samples) was discarded as a burn-in period and the remainder was thinned by 19 out of 20 samples to avoid autocorrelation. We checked the convergence of the model using Rhat (<1.1), Monte Carlo standard error (<10% of the posterior standard deviation), and effective sample size (10% or more of the total). Then we calculated (1) how many times the model yielded the first-ranked (smallest) WAIC and (2) the mean WAIC of 100 runs. In all cases we modeled, the best models in terms of (1) were also the best in terms of (2) (see tables S1 and S2). We merged all posterior distributions of 100 models for each parameter estimate and calculated the expected a posteriori (EAP) estimates and 95% credible intervals. The EAP corresponded to the mean value of the posterior distribution. Table S1 (for reach frequency) and table S2 (for food intake amount) show the list of models, EAPs, 95% credible intervals of posterior distributions, and mean WAICs. We conducted model construction and estimation of parameter distributions using R [7] (version 3.4.4) and accompanying packages; brms [8] (version 2.5.0), loo [9] (2.0.0), Rcpp [10] (0.12.19), and rstan [11] (2.17.3). For calculation of WAIC from the posterior distributions, we used source code developed by Kentaro Matsuura [http://statmodeling.hatenablog.com/entry/calc-waic-wbic].

## *Statistical analysis using Bayesian multilevel models for subjective ratings (general deliciousness and apprehensiveness about diet quantity)*

We assumed a normal distribution for the error structure of the data of subjective ratings. To access whether reach frequency or total food intake predicts subjective rating of general deliciousness, we designed the model as below.

$$\begin{aligned} X\sim Normal\left( \beta_{0}+\beta_{1}\times explanatory variable+\frac{r_{pair}}{r_{individual}} \right)\#\left( 3 \right) \end{aligned}$$

If estimated value for $\beta_{1}$ is positive, the subjective rating of general deliciousness is positively predicted by reach frequency or total food intake.

To access whether apprehensiveness about diet quantity was different by sex, we designed the model as below.

$\begin{aligned} X\sim Normal\left( \beta_{0}+\beta_{1}\times sex+\frac{r_{pair}}{r_{individual}} \right)\#\left( 4 \right) \end{aligned}$ The coefficient $\beta_{1}$ indicates the difference in females compared to males; a positive estimated value for $\beta_{1}$ indicates that the response variable *X* was larger in females compared to males. We conducted MCMC sample generation and model comparison in the same way as statistical analysis for reach frequency and food intake amount (see above).

## *State-space modeling for the weight of potato chips per reach*

To investigate the sequence of the weight of potato chips per reach, we adopted a state-space modeling approach, which enables us to estimate the stochastic process that generated the observed sequential data (see [12] for review). In a state-space model, observed values are assumed to be sampled from probability distributions called states. In the model we adopted, states can vary in each time step and a particular state in time unit *n* was assumed to be a function of the state in time unit *n*−1 (i.e. a Markov process). Specifically, we assumed that the observed weight of potato chips in the *n*th reach $Y_{n}$ was sampled from a latent state of a normal distribution with mean $\mu_{n}$, which was in turn assumed to be sampled from the state in time unit *n*−1, whose mean was $\mu_{n-1}$ plus fixed value of *trend*. The maximum *n* was 23 because the highest reach frequency value in *Solo* condition was 23. We also modeled the correlation within an individual as a random intercept $r_{i, n}$ that was assumed to be generated from a normal distribution with mean = 0. Then the differences between *Solo* versus *Visible Pair* $diff_{Visible Pair, n}$ and *Invisible Pair* $diff_{Invisible Pair, n}$were modelled as a Cauchy distribution, assuming that the differences could vary with time unit (*n*th reach). We thus developed the model as follows:


$$\begin{aligned} Y_{Solo, n} \sim Normal\left( \mu_{n}+r_{i, n}, \sigma_{Y} \right)\#\left( 5 \right) \end{aligned}$$

$$\begin{aligned} Y_{Visible Pair, n} \sim Normal\left( \mu_{n}+r_{i, n}+diff_{Visible Pair, n}, \sigma_{Y} \right)\#\left( 6 \right) \end{aligned}$$

$$\begin{aligned} Y_{Invisible Pair, n} \sim Normal\left( \mu_{n}+r_{i, n}+diff_{Invisible Pair, n}, \sigma_{Y} \right)\#\left( 7 \right) \end{aligned}$$

$$\begin{aligned} \mu_{n} \sim Normal\left( \mu_{n-1}+trend, \sigma_{\mu} \right)\#\left( 8 \right) \end{aligned}$$

$$\begin{aligned} diff_{Visible Pair, n} \sim Cauchy\left( diff_{Visible Pair, n-1}, \sigma_{diff_{Visible Pair}} \right)\#\left( 9 \right) \end{aligned}$$

$$\begin{aligned} diff_{Invisible Pair, n} \sim Cauchy\left( diff_{Invisible Pair, n-1}, \sigma_{diff_{Invisible Pair}} \right)\#\left( 10 \right) \end{aligned}$$

$\begin{aligned} r_{i, n} \sim Normal\left( 0, \sigma_{r} \right)\#\left( 11 \right) \end{aligned}$Therefore, when the group mean weight of potato chips in the *n*th reach is smaller in *Visible Pair* or *Invisible Pair* condition, the distribution of the corresponding coefficient $diff_{Visible Pair, n}$ or $diff_{Invisible Pair, n}$should be less than zero. We generated the posterior distributions of the parameters by MCMC sampling from 4 chains of 5,000 iterations for each, with the first 1,000 iterations discarded as burn-in periods and the remainder thinned by 4 out of 5 samples to avoid autocorrelation.

To detect the effect of subjective evaluation apprehension on the weight of potato chip per reach, we introduced the corresponding term $\beta\times score$ ($score$ was converted to a minimum of 0 and a maximum of 1) to the formula (6) and (7) as below:

$$\begin{aligned} Y_{Visible Pair, n} \sim Normal\left( \mu_{n}+r_{i, n}+diff_{Visible Pair, n}+\beta_{Visible Pair, n}\times score_{i}, \sigma_{Y} \right)\#\left( 12 \right) \end{aligned}$$

$$\begin{aligned} Y_{Invisible Pair, n} \sim Normal\left( \mu_{n}+r_{i, n}+diff_{Invisible Pair, n}+\beta_{Invisible Pair, n}\times score_{i}, \sigma_{Y} \right)\#\left( 13 \right) \end{aligned}$$

$$\begin{aligned} \beta_{Visible Pair, n} \sim Normal\left( \beta_{Visible Pair, n-1}, \sigma_{\beta_{Visible Pair}} \right)\#\left( 14 \right) \end{aligned}$$

$$\begin{aligned} \beta_{Invisible Pair, n} \sim Normal\left( \beta_{Invisible Pair, n-1}, \sigma_{\beta_{Invisible Pair}} \right)\#\left( 15 \right) \end{aligned}$$

Therefore, when evaluation apprehension predicts the weight of potato chips in the *n*th reach in *Visible Pair* or *Invisible Pair* condition, the distribution of the corresponding coefficient $\beta_{Visible Pair, n}$ or $\beta_{Invisible Pair, n}$ should be above or below zero.

To examine the differences between the results of the weighing and tasting experiments, we set the weight trajectory of the weighing of 2,322 pieces by two naïve subjects as a baseline and estimated the differences between that baseline and the *Solo*, *Visible Pair*, and *Invisible Pair* conditions. The model component described as Equation (5) was thus modified as follows:

$$\begin{aligned} Y_{Weighing, n} \sim Normal\left( \mu_{n}+r_{i, n}, \sigma_{Y} \right)\#\left( 16 \right) \end{aligned}$$

$$\begin{aligned} Y_{Solo, n} \sim Normal\left( \mu_{n}+r_{i, n}+diff_{Solo, n}, \sigma_{Y} \right)\#\left( 17 \right) \end{aligned}$$

$$\begin{aligned} diff_{Solo, n} \sim Cauchy\left( diff_{Solo, n-1}, \sigma_{diff_{Solo}} \right)\#\left( 18 \right) \end{aligned}$$

The other model components are described as Equations (6)–(11). We generated the posterior distributions of the parameters by MCMC sampling from 4 chains of 10,000 iterations for each, with the first 2,000 iterations discarded as burn-in periods, and the remainder thinned by 9 out of 10 samples to avoid autocorrelation.

## *Procedures for random sampling simulation from the distribution of potato-chip weights and state-space modelling*

We generated 60 trajectories of approximately 30 grams of potato chips by random sampling simulation from the weight distribution of 8,075 potato chips. To examine the differences between the results of the tasting experiments, we set the simulated weight trajectory as a baseline and estimated the differences between that baseline and the *Solo*, *Visible Pair*, and *Invisible Pair* conditions. The model component described as Equation (16) was thus modified as follows:

$$\begin{aligned} Y_{Random Sampling, n} \sim Normal\left( \mu_{n}+r_{1,n}, \sigma_{Y} \right)\#\left( 19 \right) \end{aligned}$$

The other model components are described as Equations (6)–(11), (17), and (18). We generated the posterior distributions of the parameters by MCMC sampling from 4 chains of 20,000 iterations for each, with the first 5,000 iterations discarded as burn-in periods, and the remainder thinned by 9 out of 10 samples to avoid autocorrelation.

## *Fitting a skew-Gaussian model to the weight distribution of potato chips*

To measure the skew of the observed weight distribution of 8,075 potato chips, we fitted a skew-Gaussian model to the weight distribution and estimated the mean, sigma and skewness parameters. Posterior distributions of the parameters were obtained by MCMC sampling from 4 chains of 2,000 iterations, with the first 1,000 iterations discarded as burn-in periods. The EAPs and 95% credible interval of posterior distributions of the parameters were 0.74 (0.73–0.75) for the mean, 0.50 (0.50–0.51) for the sigma, and 53.1 (48.3–58.1) for the skewness.

## *Posterior predictive check for the statistical models*

To verify the validity of the statistical models, we conducted a posterior predictive check using the best models [13]. For the model of reach frequency (figure 2A left, table S1), total food intake (figure 2A right, table S2), apprehensiveness of diet quantity (figure S2, table S3), subjective rating for general deliciousness (table S4), we generated 100 datasets from the first run (i.e. seed = 1) of the best models and checked whether the simulated datasets were similar to the actual datasets in terms of means and variances (figure S3A, S3C). For the state-space models for weight of potato chips per reach (figure 2B, 3B, 3C), we generated 100 datasets from the posterior distributions of the model parameters and successfully replicated the means and variances of the original dataset (figure S3B).

**Supplementary references**

1. Garner DM, Olmstead MP, Bohr Y, Garfinkel P. 1982 The eating attitudes test: Psychometric features and clinical correlates. *Psychol. Med.* **12**, 871–878. (doi:10.1017/S0033291700049163)

2. Mukai T, Crago M, Shisslak CM. 1994 Eating attitudes and weight preoccupation among female high school students in Japan. *J. Child Psychol. Psychiatry.* **35**, 677–688. (doi:10.1111/j.1469-7610.1994.tb01213.x)

3. Nicholls MER, Thomas NA, Loetscher T, Grimshaw GM. 2013 The flinders handedness survey (FLANDERS): A brief measure of skilled hand preference. *Cortex* **49**, 2914–2926. (doi:10.1016/j.cortex.2013.02.002)

4. Okubo M, Suzuki H, Nicholls MER. 2014 A Japanese version of the FLANDERS handedness questionnaire. *Japanese J. Psychol.* **85**, 474–481. (doi:10.4992/jjpsy.85.13235)

5. Ogura Y, Matsushima T. 2011 Social facilitation revisited: Increase in foraging efforts and synchronization of running in domestic chicks. *Front. Neurosci.* **5**, 1–12. (doi:10.3389/fnins.2011.00091)

6. Watanabe S. 2010 Asymptotic equivalence of Bayes cross validation and widely applicable information criterion in singular learning theory. *J. Mach. Learn. Res.* **11**, 3571–3594.

7. R Core Team. 2018 R: A language and environment for statistical computing.

8. Bürkner P-C. 2017 brms: An R package for Bayesian multilevel models using Stan. *J. Stat. Softw.* **80**, 1–28.

9. Vehtari A, Gabry J, Yao Y, Gelman A. 2018 loo: Efficient leave-one-out cross-validation and WAIC for Bayesian models.

10. Eddelbuettel D, François R, Allaire J, Ushey K, Kou Q, Russel N, Chambers J, Bates D. 2011 Rcpp: Seamless R and C++ integration. *J. Stat. Softw.* **40**, 1–18.

11. Stan Developmental Team. 2018 RStan: the R interface to Stan.

12. Patterson TA, Thomas L, Wilcox C, Ovaskainen O, Matthiopoulos J. 2008 State-space models of individual animal movement. *Trends Ecol. Evol.* **23**, 87–94. (doi:10.1016/j.tree.2007.10.009)

13. Kruschke JK. 2013 Posterior predictive checks can and should be Bayesian: Comment on Gelman and Shalizi, ‘Philosophy and the practice of Bayesian statistics’. *Br. J. Math. Stat. Psychol.* **66**, 45–56. (doi:10.1111/j.2044-8317.2012.02063.x)

**Supplementary figures and tables**

**Figure S1. Mere presence of co-eater did not change rating scores for the flavor of the potato chips.**

Group mean ± SEM of subjects’ evaluations of the potato chips (range: 0 – 100). (A) General deliciousness, (B) sweetness, (C) saltiness, (D) sourness, (E) bitterness, (F) umami, (G) strength, (H) texture, and (I) fillingness. The three conditions yielded no significant difference on any of the 9 dimensions.

**Figure S2. Apprehensiveness about quantity of daily diet in males and females.**

Group mean ± SEM of subjects’ evaluations of apprehensiveness about quantity of daily diet (range: 0 – 100) by sex. For statistical analysis, see table S3.

**Figure S3. Posterior predictive checks for statistical models.**

(A) Means (X axis) and SDs (Y axis) of the generated posterior predictive samples and actual data. The left panel represents the model for reach frequency (figure 2A left panel; table S1) and the right panel represents the model for total food intake (figure 2A right panel; table S2).

(B) Means of actual data and means of generated posterior predictive samples for the model shown in Equations (5)–(11) in which the *Solo* condition was set as the baseline (top panel), from the model shown in Equations (6)–(11), (16)–(18) in which the weighing experiment was set as the baseline (middle panel), and from the model shown in Equations (6)–(11), (17)–(19) in which the random sampling was set as the baseline (bottom panel).

(C) Means (X axis) and SDs (Y axis) of the generated posterior predictive samples and actual data. The left panel represents the model for apprehensiveness of diet quantity (figure S2; table S3) and the right panel represents the model for subjective rating for general deliciousness (table S4).

**Table S1. WAICs and estimated coefficients of variables for 18 models designed for reach frequency.**

The models were sorted in descending order of how many times the model yielded the smallest WAIC in 100 runs (“WAIC_1st” column). When “WAIC_1st” was the same value, the models were sorted in ascending order of mean of WAIC in 100 runs (“WAIC_mean” column). The expected a posteriori (EAP) estimates and 95% credible intervals (in square brackets) are shown for each explanatory variable. β_0_ indicates intercept. β_1_ indicates sex variable; male was assigned to be 0 compared with female. β_2_ indicates condition variable; *Solo* was assigned to be 0 compared with *Visible Pair* (upper row in each cell) and *Invisible Pair* (lower row). β_3_ indicates session variable; 1st session was assigned to be 0 compared with 2nd (upper row) and 3rd session (lower row). We also considered the models that included interactions; β_4_ indicates *sex* × *condition* interaction, β_5_ indicates *sex* × *session* interaction, β_6_ indicates *condition* × *session* interaction and β_7_ indicates *sex* × *condition* × *session* interaction.

The model that included *sex*, *condition* and *session* variables and included no interaction yielded the smallest WAIC most frequently. The mean WAIC in 100 runs was also the smallest among the 18 models we considered. Thus, we concluded that this model was the best one. The best model indicates that (1) reach frequency was lower in females compared to males; (2) reach frequency was higher in the *Visible Pair* and *Invisible Pair* conditions compared to the *Solo* condition; and (3) reach frequency was higher in the 3rd session compared to the 1st session, although the 95% CI of the coefficient included 0 at the lower bound.

**Table S2. WAICs and estimated coefficients of variables for 18 models designed for total food intake.**

The models were sorted in descending order of how many times the model yielded the smallest WAIC in 100 runs (“WAIC_1st” column).

The model that included *sex* yielded the smallest WAIC most frequently and the mean of WAIC in 100 runs was also the smallest. Thus, we concluded that this model was the best one. The best model indicates that food intake amount was lower in females compared to males and group differences between *Solo* and *Visible Pair* or *Invisible Pair* conditions were negligible.

**Table S3. Apprehensiveness about quantity of daily diet was higher in females than males.**

To examine whether the apprehensiveness about diet quantity is different by sex, WAICs and estimated coefficients of variables were calculated. Models with and without the sex variable were compared. The model with *sex* yielded smaller WAIC in all 100 runs.

**Table S4. Subjective rating for general deliciousness did not predicted by reach frequency or total food intake.**

To examine whether the subjective rating for general deliciousness is predicted by (A) reach frequency or (B) total food intake, WAICs and estimated coefficients of variables were calculated. Models with and without the sex variable were compared. In both cases, null model yielded smaller WAIC in most runs.

**Table S5. Frequency of exercise, diet restrictions regarding quantity and quality do not predict reach frequency.**

To examine the degree to which subjects’ concerns about their body shapes and diet-related behavior predicted reach frequency, WAICs and estimated coefficients of variables were calculated. (A) Models with and without the variable for exercise frequency were compared. The model without the variable for exercise frequency yielded the smallest WAIC most frequently. (B) Models with and without the variable for apprehensiveness about diet quantity were compared. The model without the variable for apprehensiveness about diet quantity yielded the smallest WAIC in all 100 runs. (C) Models with and without the variable for apprehensiveness about diet quality were compared. The model without the variable for apprehensiveness about diet quality yielded the smallest WAIC in all 100 runs.
